# Improving health systems performance in low- and middle-income countries: a system dynamics model of the pay-for-performance initiative in Afghanistan

**DOI:** 10.1093/heapol/czx122

**Published:** 2017-09-23

**Authors:** O Alonge, S Lin, T Igusa, D H Peters

**Affiliations:** 1 Department of International Health, Johns Hopkins Bloomberg School of Public Health, 615 N Wolfe Street, E8622, Baltimore, MD 21205, USA and; 2 Department of Civil Engineering, Johns Hopkins University, 3400 N Charles Street, Baltimore, MD 21218, USA

**Keywords:** Performance-based financing, implementation, system dynamics model, health services delivery, Afghanistan, low- and middle-income countries

## Abstract

System dynamics methods were used to explore effective implementation pathways for improving health systems performance through pay-for-performance (P4P) schemes. A causal loop diagram was developed to delineate primary causal relationships for service delivery within primary health facilities. A quantitative stock-and-flow model was developed next. The stock-and-flow model was then used to simulate the impact of various P4P implementation scenarios on quality and volume of services. Data from the Afghanistan national facility survey in 2012 was used to calibrate the model. The models show that P4P bonuses could increase health workers’ motivation leading to higher levels of quality and volume of services. Gaming could reduce or even reverse this desired effect, leading to levels of quality and volume of services that are below baseline levels. Implementation issues, such as delays in the disbursement of P4P bonuses and low levels of P4P bonuses, also reduce the desired effect of P4P on quality and volume, but they do not cause the outputs to fall below baseline levels. Optimal effect of P4P on quality and volume of services is obtained when P4P bonuses are distributed per the health workers’ contributions to the services that triggered the payments. Other distribution algorithms such as equal allocation or allocations proportionate to salaries resulted in quality and volume levels that were substantially lower, sometimes below baseline. The system dynamics models served to inform, with quantitative results, the theory of change underlying P4P intervention. Specific implementation strategies, such as prompt disbursement of adequate levels of performance bonus distributed per health workers’ contribution to service, increase the likelihood of P4P success. Poorly designed P4P schemes, such as those without an optimal algorithm for distributing performance bonuses and adequate safeguards for gaming, can have a negative overall impact on health service delivery systems.

Key Messages

- This study illustrates the application of an innovative modelling approach (systems dynamic modelling) for exploring causal relationships of complex interventions within dynamic health systems.
- It provides quantitative results to inform the theory of change of supply-side pay-for-performance (P4P) intervention, and show the impact of various implementation scenarios and design choices on P4P effectiveness.
- Optimal P4P design and implementation features for improving performance of health systems in low-and-middle income countries include prompt disbursement of adequate levels of performance bonus distributed per health workers’ contribution to service.
- Other distribution algorithms (such as equal allocation or allocations proportionate to salaries) and failure to mitigate gaming issues could result in health services quality and volume levels that are substantially lower, sometimes below baseline level.

## Introduction

Pay for performance (P4P) has been increasingly used as a health systems strengthening strategy in low- and middle-income countries (LMICs) (Petersen *et al.* 2006; Oxman and Fretheim 2009; Meesen *et al.* 2011; Eijkenaar 2012). It is particularly attractive to donor organizations that have traditionally invested in input-based programme activities while recognizing the agency problem that exists with such an approach (Oxman and Fretheim 2009; Meesen *et al.* 2011). The agency problem arises when providers (agents), under obligation to use resources in the interest of other individuals or groups, divert the resources for their own interest because of the disparity in information among the different parties. ‘P4P’ in this context allows a funder (principal) to place monetary incentives on the performance of providers (agents) measured as improved health services outputs and/or outcomes. The monetary incentives are used to align the funder’s objectives around improving health outputs and outcomes with the priorities of service providers involved in the actual delivery of health services.

Empirical evidence supporting the effectiveness of P4P in improving health service delivery in LMICs is mixed (Petersen *et al.* 2006; Oxman and Fretheim 2009; Eijkenaar 2012). Whereas studies from countries such as Cambodia, Rwanda, and Haiti showed that P4P led to increased utilization of maternal and child health (MCH) services (Schwartz and Bhushan 2004; Eichler and Levine 2009; Basinga *et al.* 2011), other studies indicated that P4P did not result in improved services delivery (Eichler and Levine 2009; Ssengooba *et al.* 2012). Although all these studies acknowledged the importance of context and implementation activities on the impact of P4P, none studied the interacting effects of these factors exclusively.

Since 2003, public provision of health services in Afghanistan has been largely provided through donor financed contracts with non-governmental organizations (NGOs) (Alonge *et al.* 2015). Whereas the health services delivery contracts led to improvements in utilization and quality of general ambulatory care, utilization of critical MCH services remained relatively low (Belay 2010). In 2010, skilled birth attendance (SBA) at delivery was only 19% and the country’s maternal mortality ratio of 584 per 100 000 live births is the highest outside of sub-Saharan Africa (APHI/MoPH *et al.* 2011).

To increase coverage and quality of key MCH services, the Afghanistan Ministry of Public Health (MoPH), with support from the World Bank, decided to test a P4P intervention applied at the health facility level in 9 out of the 32 provinces of the country in September 2010 (Engineer *et al.* 2016). The goal of this intervention was to improve the volume and quality of key MCH services at lower-level facilities with the hope that such improvements will lead to better coverage of quality MCH services and reduction in maternal and under-5 mortality. Performance bonuses, additional to regular budgets, were awarded to NGOs managing health facilities that performed above baseline levels in nine streams of health services. The streams of services include antenatal care for pregnant women, SBA at delivery and immunization services. The P4P intervention was implemented between September 2010 and December 2012.

The volume data that were used to determine bonuses was based on self-reported information collected at the health facility level through the Health Management Information System. Prior to payment, a random sample of the volume data was verified by independent monitors through home visits with the service beneficiaries. The performance bonuses were also adjusted based on quality scores, giving more weight to higher quality scores before authorizing final payments. The quality scores were based on independent assessments of structural quality of health facilities, including assessments of health facility equipment, infrastructure functionality and drug availability.

Although the performance payments were authorized quarterly upon verification, significant lags existed between the time of authorization and disbursement of bonuses to frontline health workers (Dale 2014). There was also no standard approach for distributing the performance bonuses among health workers within a health facility (Dale 2014). Health facilities managers distributed the performance bonuses in one of three ways: equal bonuses to all staff members; bonuses proportional to health worker salaries; and bonuses based on the direct contributions of the individual health workers to services that triggered the P4P payments. There were initial concerns about the small size of the performance bonus; however, these concerns were promptly addressed by raising the amount of payment per unit service (Engineer *et al.* 2016).

The theory of change developed by the MoPH and other stakeholders for describing the pathway for effectiveness of the P4P intervention in Afghanistan was not explicitly stated (Engineer *et al.* 2016). It was implicit that monetary incentives would have an effect on the extrinsic motivation of health workers. Studies on motivation in workplaces have suggested that extrinsic motivation, unlike intrinsic motivation, may be externally regulated and responsive to both monetary and non-monetary incentives (Deci and Ryan 2002; Gagne and Deci 2005; Pomp 2010). The MoPH theorized that providing rewards on volume and quality of service directly to frontline health workers (as supplements to their monthly salaries) would motivate these workers to improve their performance in measurable terms. The pathway for attracting eligible clients to the facility-based services to increase the quantity of service was however not explicitly defined (Engineer *et al.* 2016). Hence, the P4P intervention in Afghanistan was largely supply based.

Initial findings from a cluster-randomized study conducted between August 2010 and December 2012 to assess the effectiveness of the P4P intervention in Afghanistan showed no significant improvement in the coverage and overall quality of MCH services despite the disbursement of the performance bonuses (Engineer *et al.* 2016). The evaluation cited poor implementation of the scheme and lack of attention to demand side factors as potential reasons for the lack of effect. Some of the underlying implementation issues suggested are: the lag between authorization and disbursement of payment, initial complaint about the size of the bonuses among health workers, and the fact that only 38% of health workers in eligible health facilities reported receiving performance bonuses despite disbursement to all such facilities (Dale 2014). Another potential issue is likelihood of some actors responding to the performance bonuses in perverse ways (Glasziou *et al.* 2012; Woolhandler *et al.* 2012). Such perverse responses may include ‘gaming’ or manipulation of the verification/authorization process to obtain performance bonuses without improving the quantity and quality of services delivery (Glasziou *et al.* 2012; Woolhandler *et al.* 2012). The cluster-randomized study design is intended to demonstrate whether an effect was produced, but is limited in its ability to examine how these various implementation issues influence the intervention outcomes.

A system dynamics modelling (SDM) approach can be used to capture dynamic, non-linear relationships within a complex system, such as those observed in implementing the P4P intervention within health facilities in Afghanistan, and can provide insights as to how various aspects of inter-connected processes work together from a holistic perspective (Forrester 1969; Hirsch and Miller 1974). SDM has been previously shown to be well suited for analysis of health services delivery processes within health facilities (Hirsch and Miller 1974; Royston *et al.* 1999; Homer and Hirsch 2006; Meker and Barlas 2013). The objective of this article is to use SDM to explore and test the implicit theory of change for the implementation of the supply side P4P scheme in Afghanistan. The model is used to illustrate the dynamic changes in quality and volume of health services at the primary health facility level under the P4P intervention. In addition, the model is used to assess the potential impact of poor implementation and gaming of the P4P scheme on the volume and quality of services. We hope that the results of this study would demonstrate the significance of effective implementation on the success or failure of P4P interventions, and suggest pathways and approaches for improving the design and implementation of P4P and similar programmes in LMICs.

## Methods

The SDM (model) process used in this paper consists of two basic stages. The first stage is the development of causal loop diagrams (CLDs), which is a visual representation of a system showing the main variables and the causal relationships between them (Richardson and Pugh 1981; Homer and Hirsch 2006). The second stage is the construction of a quantitative stock-and-flow model that incorporates the qualitative relationships represented in the CLD. In the following section, we describe this two-stage process for modelling basic operations at the primary health facility level in Afghanistan, followed by a description of how we incorporate the additional relationships and variables associated with the P4P intervention.

### SDM for basic operations at the health facility level in Afghanistan

The data used for the estimating the model were based on the Afghanistan national health facility survey conducted in 2012 (Engineer *et al.* 2016). The health facility survey data contains multiple variables that are related to the supply of health services. In creating the initial CLD, we use the following three aggregated variables:

- Volume of services, based on the average number of clients attending a health facility in a month.
- Quality of services, conceived as capabilities of a health facility to provide services, which can accumulate or deplete over time. It is based on various measures of clinical processes of care, including health workers’ knowledge and performance on clients’ assessments, time spent with patients; structural measures on availability of resources including equipment and drugs; and clients’ perception of quality (Peters *et al.* 2007).
- Revenue, quantified by the total money provided to a health facility every month to cover both capital and operational expenditures. This includes funds for wages and salaries.

**Figure 1.**
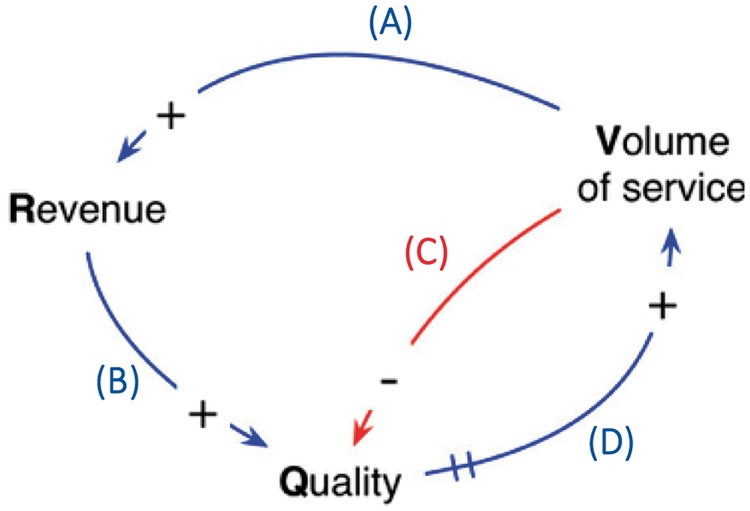
CLD for basic operations at the health facility level

The causal relationships between these variables are summarized in Figure 1.

There are four causal relationships, (A–D), among the three variables (revenue, quality and volume of service) as shown in Figure 1. Positive causality is indicated in all the arrows except for the arrow from volume to quality, which represents the negative causal relationship (C). There is a time delay between quality and volume of service which is indicated by the double line shown near the bottom of Figure 1 through arrow (D). Detailed explanation on the causal relationships is included in the Supplementary Appendix S1.

Two closed loops can be seen in this CLD. The outer loop consists of three positive causal relationships, (A), (B) and (D), connecting the three variables counter-clockwise. In system science language, this is known as a reinforcing loop, whereby increase in a variable result in an increase in each subsequent variable in the loop (Richardson and Pugh 1981). If left unchecked, this series of positive causal relations would result in unlimited increases in all the variables involved. There is, however, a second smaller loop, between quality and volume, in which the positive causal relationship (D) is counteracted by a negative causal relationship (C). This is known as a balancing loop; it acts as the check that prevents unbounded increases in the variables (Richardson and Pugh 1981).

Using this CLD, we proceed with the second stage in the modelling process, which is the construction of the quantitative stock-and-flow model. In general, there are three types of variables in stock-and-flow models: ‘stocks’, which represent quantities that are accumulated or depleted over time; ‘rates’, which are flows that increase or decrease the levels of the stocks; and ‘auxiliary variables’, which are constants that parameterize the model (Richardson and Pugh 1981; Homer and Hirsch 2006). The auxiliary variables are often related to exogenous influences, and can affect both the stocks and rates. The relationships between the stocks and rates are formalized using differential equations and other mathematical operations (Richardson and Pugh 1981; Homer and Hirsch 2006). Ideally, long-duration time-series data are used to study the long-term equilibrium states of the system. When such data is not available, simulated data can still provide useful information for policy recommendations (Homer and Hirsch 2006).

**Figure 2.**
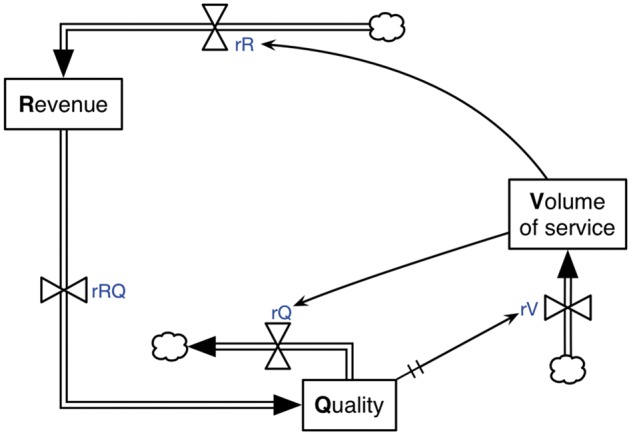
Stock-and-flow diagram for basic operations at the health facility level in Afghanistan

In our stock-and-flow model, we use the three aggregated variables from the CLD (revenue, quality and volume of service) as the ‘*stock variables’*. These variables are shown in boxes in the stock-and-flow diagram in Figure 2.

The causal relationships (A–D) in the CLD are represented in the stock-and-flow diagram (Figure 2) through rate-controlled flows as explained in the following steps: (1) we begin with the positive causal relationship from volume to revenue. At the top of Figure 2, the broad arrow with doubled line represents a flow into the revenue stock. The double triangle is a valve that regulates the rate of this flow. As the volume of service increases, the rate of flow at the valve increases, generating more revenue. This relationship between volume and rate of flow is indicated by the thin curved arrow pointing to the valve. The cloud at the top is used to indicate a source that is exogenous to our model; for this particular flow, the source would be the governmental institutions that are funding the health facilities. (2) Next, we consider the positive causal relationship from revenue to quality. This is represented by a flow directly from the revenue stock to the quality stock, shown at the left side of Figure 2. As described later in this section, ‘unitless’ quantities are used for the stock variables; this simplifies the model and allows for the flow of one stock to another. (3) The causal relationship between quality and volume is represented by the flow at the right side of Figure 2. This is a flow into volume that originates from another source; in this case, the source is the potential clients residing in the health facility catchment area. The valve that regulates this flow is connected to the quality stock variable whereby the flow into volume increases when quality also increases. (4) The only negative causal relationship in the CLD, from volume to quality, is shown at the bottom of Figure 2 above the quality variable. Here, the cloud represents a sink, which can be interpreted as a drain of the quality stock. The rate of flow into this drain is increased when the volume of service increases; this relationship is indicated by the arrow to the valve above the quality stock. In summary, there are four flows in the stock-and-flow diagram in Figure 2, each with a valve that regulates the rate of flow; these flows and corresponding valves correspond to the four causal relationships in the CLD in Figure 1.

The rates of flow in the stock-and-flow model are quantified by four ‘rate variables’, described in below:

- Recovery rate of revenue (rR), this is the rate at which revenue is provided to a health facility.
- Conversion rate of revenue to quality (rRQ), this is the rate at which revenue provided to a health facility improves the measures of quality of services.
- Depletion rate of quality (rQ), this is the rate at which the quality of services declines when the volume of service increases.
- Recovery rate of volume (rV), this is the rate at which the volume of services increases in response to an increase in the quality of service. Such a response may be delayed depending on how quickly clients perceive the change in quality and ‘spread the message’ to other clients. This time delay is explicitly incorporated into the model.

We conclude the description of the SDM by reiterating its purpose, which is to provide insights into how key implementation processes could influence outcomes of P4P interventions. The focus is on showing general trends in response to different P4P implementation approaches rather than to provide predictive results (or behaviour accuracy). The absolute values of stock variables for a modelled health facility were calibrated to the range of all possible values from real data available for 641 health facilities (in the Afghanistan national health facility survey conducted in 2012). That is, the changes in volume data with corresponding changes in quality data, for a range of health facilities in Afghanistan, were used to assume the dynamic changes in a single modelled health facility over time. To allow for a straightforward interpretation of results, we scale the ‘unitless’ stock variables from −1 to 1, with −1, 0 and 1 representing below-average, average, and above-average outcomes, respectively. The basic mathematical relationships between the stock and rate variables for the SDM of basic operations are given in the Supplementary Appendix S1 (Table SA1).

**Table 1.** Summary of simulation results for the nine P4P implementation scenarios

Group characteristics	Scenario description	Quality at equilibrium	Volume at equilibrium	Time of equilibrium (months)
1. No P4P	1.Baseline	0	0	60
2. P4P	2.P4P only	1	1	60
3. P4P with one additional feature	3a.Motivation	1	1	40
	3b.Low-level gaming	0	0	60
	3c.High-level gaming	−1	−1	20
	3d.Bonus delay	1	1	75
	3e.Low bonuses	0	0	60
4. P4P with motivation, gaming and bonus delay, with different bonus distribution strategies	4a.Equal allocation	0	−0.15	—^a^
	4b.Proportionate to salaries	−1	−1	60
	4c.Proportionate to services	1	1	60

a There is no equilibrium at 100 months, so the quality and volume at 100 months is shown.

The quantitative stock-and-flow model were implemented using MATLAB and Simulink computer programming platform (Mathworks 2015).

### Incorporation of the P4P intervention into the SDM

In this section, we describe the relationships and variables that are added to the model to represent the P4P intervention and its impact on the health facility system. As before, we follow a two-stage process; in the first stage, we extend the CLD in Figure 1 to include the variables and causal relationships associated with the intervention. In the second stage, the relationships in the extended CLD are used to expand the stock-and-flow model from Figure 2.

**Figure 3.**
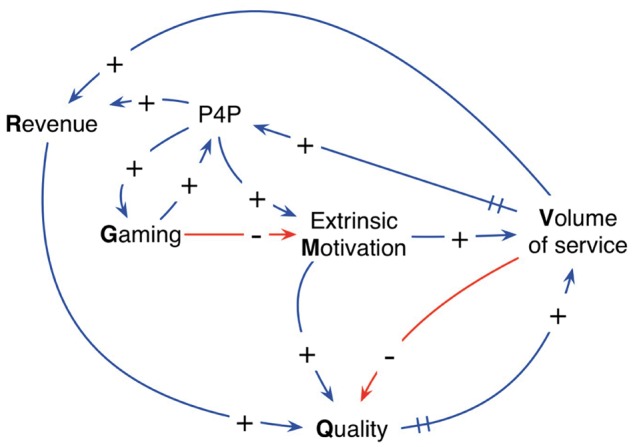
Extended CLD showing the effects of P4P bonuses on operations at the health facility level

Given the background review for this study, it is necessary to explicitly add three variables to the original CLD to create the extended CLD: the ‘P4P bonus’ along with ‘extrinsic motivation’ and ‘gaming’. Figure 3 shows these variables and their causal relationships.

By following the arrows in the CLD, increased volume results in increased P4P bonuses, which propagates to increased revenue, extrinsic motivation and the tendency for gaming. Increases in extrinsic motivation cause an increase in both volume and quality of service. Finally, increases in gaming will result in further increases in P4P bonuses as well as negative effects on extrinsic motivation. Gaming introduces several negative feedback loops as shown in Figure 3, such as the one from gaming to extrinsic motivation that successively impacts volume and P4P before returning to gaming. The effects of these negative feedback loops will be apparent with the stock-and-flow model results presented in the next section.

**Figure 4.**
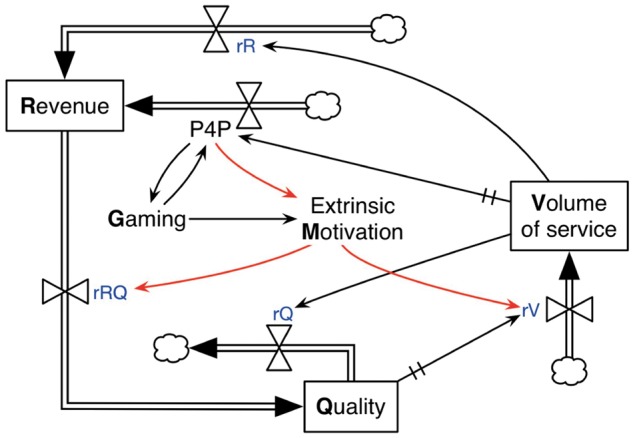
Stock and flow diagram showing the impact of P4P bonuses on operations at the health facility level in Afghanistan

Using the extended CLD in Figure 3, we proceed with expanding the quantitative stock-and-flow model from Figure 2. We incorporate P4P bonuses using a ‘rate variable’ and represent extrinsic motivation and gaming as ‘auxiliary variables’, as explained in the following descriptions (Figure 4)

#### P4P

This is a measure of the performance bonuses, which is directly related to the number of new clients at a health facility in a previous time cycle. Higher values of P4P results in increased flow into revenue (as indicated by the arrow from P4P to revenue in Figure 4). Although P4P could move bi-directionally (i.e. from a lower to a higher value and vice versa), it is never negative. That is, health facilities are never penalized if their volume of services falls below their baseline levels.

#### Extrinsic motivation

This is the extent to which health workers agree that certain aspects of their job such as time spent with clients were motivated by the performance bonus (Dale 2014). It is impacted by P4P and it controls the conversion rate of revenue to quality and the recovery rate of the volume of service as shown in Figure 4. It is assumed that some baseline motivation exists among health workers irrespective of the P4P intervention; hence, extrinsic motivation always assumes a non-negative value (Deci and Ryan 2002; Gagne and Deci 2005).

#### Gaming

This variable was assigned a value between 0 and 1, in which gaming = 0 and gaming = 1 correspond to health facilities with no gaming and extensive gaming, respectively. It is assumed that simultaneous positive causal relationships exist between gaming and P4P, i.e. gaming leads to increased (but unwarranted) P4P bonuses, which, in turn, encourages further gaming. It is also assumed that gaming in a health facility reduces extrinsic motivation of health workers and increases the depletion rQ of service.

The mathematical relationships between these additional auxiliary and rate variables for modelling the impact of P4P bonuses at the health facility level are given in the Supplementary Appendix S1 (Table SA2).

We conclude this subsection by summarizing the main characteristics of the stock-and-flow model in Figure 4: P4P bonuses are controlled by volume of service, with a delay function in the relationship. This delay arises because P4P bonuses in a current cycle are disbursed based on the volume of service of the preceding cycle. The release of P4P bonuses results in an extra flow into revenue. P4P bonuses have a positive effect on extrinsic motivation. Extrinsic motivation increases the conversion rate of revenue to quality and the recovery rV of service. Gaming harms quality of service and extrinsic motivation, but has a positive effect on P4P bonuses. Hence, a reinforcing feedback loop exists between P4P and gaming. It is noted that if we set P4P, extrinsic motivation and gaming variables to be zero, the model reduces to the simplified model of basic operations of health facilities in Afghanistan, shown in Figure 2.

### Validity testing

We conducted a series of literature and qualitative expert reviews to establish content and conceptual validity of the variables, and the characteristics of the causal relationships among these variables, that were included in the CLDs for the Afghanistan context. Statistical tests (including regressions) were also used to explore the relationship among these variables. The CLDs were revised and simplified over several iterations based on these reviews and tests.

We also conducted formal validity tests over two major iterations during our development of the quantitative stock-and-flow models presented in this study to ensure that all model structures are valid, and behavioural patterns are consistent with general knowledge around P4P. The formal validity tests include direct structure tests; structure-oriented behavioural tests (including extreme condition testing), and limited behaviour sensitivity tests. The details of the formal validity tests are presented in the Supplementary Appendix S1; these details are organized based on a scheme proposed by Barlas (1996) and include results from our behaviour sensitivity tests (Table SA3).

### Simulation of the potential impact of various implementation issues on the P4P intervention

Using the stock-and-flow model in Figure 4, we simulated two groups of scenarios (Box 1) with the goal of developing a revised theory of change based on different implementation scenarios under the P4P intervention.

**Box 1.** P4P design and implementation scenarios

First group of scenarios**:** (1) We described *the baseline scenario* with no P4P. We then considered (2) *the simplest P4P model*, which did not include gaming, motivation driven by P4P or bonus payment delays. Then we simulated five *P4P models*, in which we added one factor at a time: (3a) *motivation*, (3b) *low-level gaming*, (3c) *high-level gaming*, (3d) *bonus payment delays*, and (3e) *low bonuses*.

Second group of scenarios: we considered a system that included motivation driven by P4P payments with the delay of bonuses, but which differed per the way in which the bonuses were distributed within the facility. The details of these models of P4P bonus distribution are described below:

(4a) Equal allocation to all staff. We theorized that the workers are likely to be motivated according to the relative size of their bonuses as compared with their earnings (Camerer and Hogarth 1999). Hence, the bonuses may only be remarkable for low wage earners (Camerer and Hogarth 1999). Under such a scenario, we assumed that P4P activates extrinsic motivation, but extrinsic motivation is only able to influence the flow leading to increased quality and not the flow leading to increased volume.

(4b) Proportionate to salaries. We assumed that the leadership of the facility will receive higher bonuses and will be more motivated than other workers because of their relatively large salaries (Camerer and Hogarth 1999). We theorize that the salaries of most rank-and-file health workers may be already small. Hence, their bonuses may be insufficient to activate their extrinsic motivation and performance (Camerer and Hogarth 1999). Moreover, the disparity in bonuses could be perceived as unfair by the rank-and-file health workers, and may undermine the intrinsic motivation of these workers (Deci and Koestner 1999). The health facility leadership is also more likely to be involved in finalizing the data upon which payments are made and may be more tempted to game (Glasziou *et al.* 2012). Hence, we assume under this scenario that P4P does not activate extrinsic motivation, thereby allowing for a scenario in which the intervention may be harmful to overall performance (Glasziou *et al.* 2012; Miller and Babiarz 2013).

(4c)* Proportionate to contribution to services that triggers payment.* Individual health workers are likely to be strongly motivated if they were recognized for their individual efforts in achieving higher levels of quality and volume of services (Camerer and Hogarth 1999; Deci and Koestner 1999; Deci and Ryan 2002), and this may be sufficient to raise the aggregate motivation of key staff within the health facility and enough to increase the flows to volume and quality. Such dynamics will, however, be contingent upon strong leadership in the health facility and the extent to which the leadership’s effort is compensated as part of this implementation scenario (van Herck *et al.* 2010; Miller and Babiarz 2013).

In each simulation, the changes in the stock variables (quality, volume, and revenue) were computed over 100 months. The initial quality was set to be average (*Q* = 0), while the initial volume was set to be slightly below average (*V* = −1/2). This volume deficit was introduced so that we could examine whether the various scenarios could correct this deficit or even increase volume to levels above average.

The first group of scenarios was simulated by turning on and off the various components of the systems dynamics model. The parameters used for each of these scenarios are shown in the Supplementary Appendix S1 (Table SA3, rows 1–3e). The second group of scenarios was modelled by changing the influences of the P4P bonuses on extrinsic motivation, and the subsequent effects of extrinsic motivation on the recovery rV and conversion rate of rRQ. These changes are highlighted in red in the diagram in Figure 4, and the parameter values that correspond to each scenario are shown in the Supplementary Appendix S1 (Table SA3, rows 4a–4c).

## Results

In this section, the simulation results for the two groups of scenarios are shown and summarized (Table 1).

**Figure 5.**
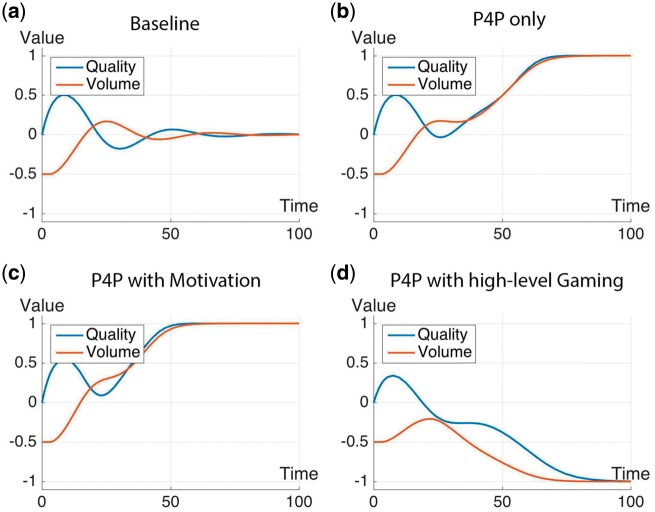
Simulation results showing changes in quality and volume of services at a health facility for: (a) Baseline, (b) P4P only, (c) P4P with motivation, and (d) P4P with high-level gaming

**Figure 6.**
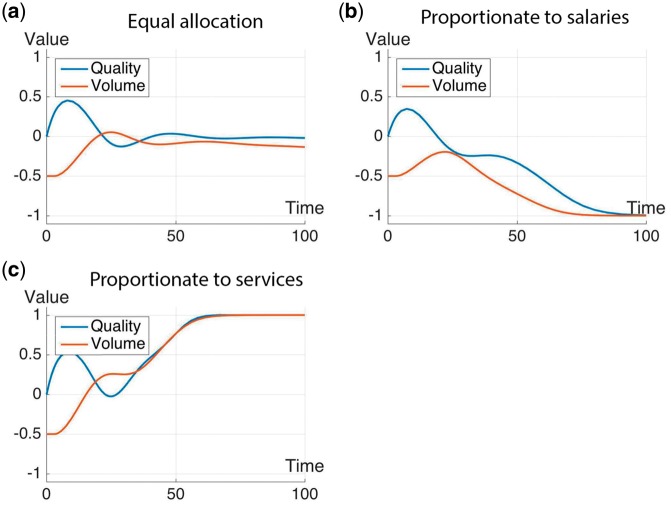
Simulation results showing changes in quality and volume of services with the following scenarios for the allocation of P4P bonuses: (a) equal allocation to all staff, (b) allocation proportionate to salaries, and (c) allocation proportionate to contribution to services that triggers payment

The time-varying plots for the quality and volume of service are shown in Figure 5 for the first group of scenarios, and in Figure 6 for the second group of scenarios.

(1) Baseline simulation (Figure 5a). For this scenario, in which there is no P4P, initial quality is at the average level (*Q* = 0) and the initial volume is slightly below average (*V* = −1/2). It can be seen in Figure 5a that quality and volume fluctuate and eventually converge to average values (*Q* = *V* = 0). If the fluctuations are examined more closely, quality would initially improve over time given adequate but fixed revenue. This improvement in quality (*Q* > 0) would result in an increment in the volume of service leading to an overextension of the capacity of the health facility (*V* > 0). As volume increases further, quality will start to decline towards the baseline value. The decline in quality could fall below baseline level when volume of service is above capacity (time = 30 months). The decline in quality will eventually results in a falling of the volume of services (time = 40 months). These fluctuations continue until the system approaches equilibrium (time = 60 months) where the baseline quality in the health facility is sufficient to keep volume of services at average levels (*Q* = *V*= 0 at equilibrium).

(2) P4P only (Figure 5b). P4P increases the revenue accruing to a health facility. Hence, the introduction of P4P would allow a health facility to increase both its quality and the capacity to increase its volume of service significantly above the baseline level (*Q* = *V* = 1) at equilibrium (time = 60 months).

(3a) P4P with motivation (Figure 5c). The simulation result for this scenario is similar to that of Scenario 2 (Q and *V* = 1), but with attainment of the higher performance at a faster rate (equilibrium at 40 months).

(3b) P4P with low-level gaming (like Figure 5a). Here, the effect of P4P is counteracted by gaming; hence, the plot is like the baseline results in Figure 5a and an additional plot is not shown. Since the gaming is at a low level, quality and volume of service does not go below the baseline levels.

(3c) P4P with high-level gaming (Figure 5d). The negative effects of gaming are stronger than in the preceding case. The consequence is that performance falls to a much lower level relative to baseline (*Q* and *V* = −1); furthermore, this negative state is attained at a much faster rate (20 months).

The results for the next two scenarios are like some of the preceding simulation results, so additional plots are not shown.

(3d) Delay between authorization and disbursement of P4P payments to health workers (like Figure 5b). This delay is not serious enough to affect the beneficial impact of P4P, and the results showed relatively high levels of quality and volume (*Q* and *V* = 1). The difference from Scenario (2) is that equilibrium was attained at a slower rate when delay is factored in (time = 75 months).

(3e) Low-level bonuses (like Figure 5a). Here, the impact of the P4P bonuses was diminished, and the results become similar to those for the model under basic operation of a health facility without P4P (*Q* = *V* = 0 at 60 months).

Finally, the simulation results for the three bonus distribution strategies (second group of scenarios) are described in Figure 6.

(4a) Bonus allocated equally to all staff (Figure 6a). Quality of service initially increases above baseline. This improvement in quality leads to an increase in volume of service, which then causes quality to decline. The decline in quality eventually leads to a decline in volume. Extrinsic motivation is not sufficiently high enough to counteract this final decrease in volume, but it does support a recovery of quality back to its baseline rate (*Q* = 0 and V = −0.15 at 100 months).

(4b) Bonus allocation proportionate to salaries (Figure 6b). Both quality and volume of service fall below baseline levels because the P4P bonuses are not sufficiently high to activate extrinsic motivation, and may be influenced by gaming, which reduces performance. Hence, both quality and volume of service reduce to equilibrium levels lower than baseline rates (*Q* and *V* = −1 at 60 months).

(4c) Bonus allocation proportionate to contribution of services that triggers payment (Figure 6c). Both quality and volume of services increase over time and are maintained at equilibrium levels higher than baseline rates. This is because the P4P bonuses are sufficient to activate extrinsic motivation, which in turn supports an increase in volume and quality of services even in the presence of gaming (*Q* and *V* = 1 at 60 months).

As a supplement to the time-plots of system performance in Figures 5a–d and 6a–c and the summary of results in Table 1, we include spreadsheets of all data points, including the initial values and ranges, for the time-plots (Supplementary Appendix S2).

## Discussion

The model results provide insights into a theory of change for the P4P intervention in Afghanistan, which can be helpful in designing similar interventions in Afghanistan and other LMICs in future. In general, the results show that P4P would likely have a beneficial effect on increasing the volume and quality of health services if properly designed. The results also show the potential for ineffectiveness. For instance, the beneficial effects of P4P may be lost if the impact of gaming is not adequately mitigated, and/or there is lack of attention to specific design issues including the adequacy of the performance bonus and the way performance bonuses are distributed among health workers. The model suggests that health facility performance may worsen under a P4P scenario (relative to a scenario without P4P) if gaming overrides health workers’ motivation or when performance bonus is not equitably distributed (with respect to service efforts that triggered the payments).

Studies have suggested that, aside from the principal-agent problem that exists between a funder and a contracted organization, an internal principal-agent problem may also exist within the contracted organization, specifically between management and frontline health workers (van Herck *et al.* 2010; Miller and Babiarz 2013). The studies indicate that timely and adequate level of incentives delivered to critical frontline health workers are important for achieving performance goals (Rosenthal and Frank 2006; van Herck *et al.* 2010; Miller and Babiarz 2013).

In Afghanistan, performance bonuses to health workers were provided centrally through contracted NGOs who in turn delivered these payments to health facilities as additional funds to their operational budget (Forrester 1969; Engineer *et al.* 2016. Some health workers were in fact not aware that performance bonuses were included as part of their health facility operational budget and monthly salaries (Engineer *et al.* 2016). The managing NGOs also had significant autonomy in deciding how the performance bonus was spent and distributed among their employees (Engineer *et al.* 2016). Given the way incentives were transmitted to health facilities and the heterogeneity in allocating bonuses, it is possible that some individual health workers who deserve the rewards and whose extrinsic motivation is critical for improving health services performance at a facility may not have received any bonus (van Herck *et al.* 2010; -->Miller and Babiarz 2013; Dale 2014 ).

The internal principal–agent problem at the health facility level may be best handled by allocating incentives proportionate to contribution to services that triggered payment because this allocation strategy would tend to promote communication with health workers on the reasons for the varying levels of bonuses (van Herck *et al.* 2010). However, such a strategy can impact team cohesion and cooperation, and may be difficult to implement (Glasziou *et al.* 2012; Miller and Babiarz 2013). Hence, it is important that officers-in-charge are independently incentivized based on their facilities’ performance since they will have to seek innovative approaches to coordinate team processes and track efforts within their health facilities (Miller and Babiarz 2013).

Although service records under the Afghanistan P4P programme were verified prior to payments, gaming cannot be ruled out given the prevalence of gaming under P4P schemes. Studies have suggested that gaming (through manipulation of service data or collusion) is common in P4P systems, even under strict accountability frameworks (Glasziou *et al.* 2012; Woolhandler *et al.* 2012). Measurement of performance under such conditions may inadvertently include a provider’s ability to game the system. It is therefore essential to ensure that the effect of P4P on extrinsic motivation of critical health workers is maximized to preserve the overall positive benefit of the scheme in the presence of gaming (Woolhandler *et al.* 2012). Based on the models from our study (Table 1), we see that such trade-offs between motivation and gaming is maximized for motivation when incentives are allocated proportionate to services that triggered the payments in Afghanistan. It is also possible that other allocation arrangements may affect these trade-offs differently given the health facility context in other settings.

Our study suggests that it may take five years or longer for the P4P system to attain equilibrium (Table 1). However, most P4P interventions are usually evaluated after only 2 3 years of operations (Eichler *et al.* 2009; Basinga *et al.* 2011; Engineer *et al.* 2016). Our simulation plots in Figures 5 and 6 indicate that, at such relatively short time intervals, the effect of the P4P may not yet be perceptible or the state of the system may still be in flux. Previous studies have also shown that many of the desirable behavioural changes revert once the incentives are removed (Lester *et al.* 2010). Hence, P4P should not be seen as conclusive in terms of approaches for strengthening health systems performance, but rather as a means to an end, with the ultimate goal of enabling organizational culture that could sustain desirable individual behaviours.

Our models suggest that low bonus levels can result in severely diminished effects of P4P at the health facility level as seen in other studies (Petersen *et al.* 2006; Lester *et al.* 2010). Some authors have suggested that efforts to determine adequate levels of performance bonuses should involve the health workers (Glasziou *et al.* 2012; Woolhandler *et al.* 2012), context-specific information on the cost and productivity of an average health worker, and the utility functions of both the health worker and managing NGO (Laffont and Tirole 1993; Salanie 2005). Although the P4P bonuses paid to health workers in Afghanistan were increased from 6–11% to 14–28% above base salaries (Engineer *et al.* 2016), there is no proof that such levels are sufficient in of themselves. In cases where baseline salaries may be already small, such an increment may not attain the expected impact without adequate consideration of the health workers’ utility function.

## Conclusion

The impact of P4P interventions depends on design choices and effective delivery strategies that target incentives to key health workers at the health facility level. Specific activities such as prompt disbursement of adequate levels of performance bonuses distributed proportionately to health workers’ contribution to service could increase the chances of success of a P4P intervention. Poorly designed P4P programmes with ill-advised rules for distributing performance bonuses that are not thoroughly vetted through a theory of change and inadequate safeguards for gaming could be harmful to health service delivery systems. Furthermore, some patience is needed to observe the success of a P4P intervention. The beneficial effects of P4P are likely not perceptible within the typical 24–36 months that is typical of most impact evaluations.

## Limitations and future work

Whereas the SDMs enabled us to explore and observe, through simulation, the potential impact of complex interactions and specific implementation activities around P4P at the primary health facility level in Afghanistan, it is limited by our assumptions and simplifications. For instance, our assumption in the CLD that high volume of service ultimately leads to decline in quality may not apply for services where quality of delivery improves with practice (e.g. complex surgical procedure). Also, we simplified quality of service as an aggregated variable that describes the capabilities of a facility to provide service. This aggregated variable may comprise of different domains of quality in a real system (e.g. structural quality, technical quality, perception of quality) and these domains may not always move in the same direction, which in turn may influence the corresponding model parameter and systems behaviour. Other simplifications such as exclusion of a potential delay in the causal link between volume of service and quality of service may have implications for P4P system behaviour in non-Afghan settings. Our models are also not exempted from omitted variable bias. For instance, the model may be missing other interactions and unintended consequences that may be important for exploring the dynamic impacts of P4P at the health facility level.

A keen topic of interest for extending the work presented herein is in consideration of the demand-side of the health system in P4P interventions. From the research perspective, demand-side analysis would provide a natural pathway towards incorporating additional economic theories into the SDM.

## Ethical approval

Ethical approval was obtained from the authors institutes.

## Funding

This study was partially supported by financial support provided by the UK Department for International Development (DFID) for the Future Health Systems Research Programme Consortium. This document is an output from a project funded by DFID for the benefit of developing countries. DFID did have any role in the design or analysis of this study. The views expressed are not necessarily those of DFID.

*Conflict of interest statement*. None declared.

## Supplementary data

Supplementary data are available at *HEAPOL* online.

## Supplementary Material

Supplementary Appendix 1

Supplementary Appendix 2
