# Supplementary material for: Improving health systems performance in low- and middle-income countries: a system dynamics model of the pay-for-performance initiative in Afghanistan
Source: Health Policy Plan. 2017 Sep 23;32(10):1417–26. doi: 10.1093/heapol/czx122 (PMC5886199; doi:10.1093/heapol/czx122)
Supplement: Supplementary Appendix 1 [file afghanistan_p4p_online_appendix_1_czx122.docx]

**ONLINE APPENDIX 1**

**Improving health systems performance in low and middle income countries: A systems dynamic model of the pay-for-performance initiative in Afghanistan**

Alonge, O, MD PhD MPH; Lin, S, PhD; Igusa, T, PhD; and Peters, DH, MD DrPH

**Explanation of causal relationships among revenue, volume and quality of services in the model**

Revenues are provided through a health services provision contract based on average cost per capita for an estimated coverage population (Alonge et al. 2015). **(A)** An increase in service volume would trigger an increase in monthly revenue. **(B)** Increases in revenue available to health facilities will allow NGOs that are managing those facilities to convert some of the revenue into capabilities (e.g. supplies, equipment and retraining) that could eventually lead to improved quality of service. **(D)** Existing clients respond to improved quality of services by encouraging other clients to attend the facility, which would result in an increase in the volume of service after a period (time delay). **(C)** In the short- to mid-term period, the capacity to deliver service at a health facility is fixed; hence, an increase in volume of service beyond a critical value or threshold during such a period would result in declining quality of service. A time delay was initially included in the causal linkage between volume and quality of service, but this was later excluded based on qualitative feedback on the basic operations primary health facility in rural Afghanistan.

**Mathematical relationships between the variables used in the models**

In this appendix, we summarize the mathematical relationships between the stock, rate and auxiliary variables that are used in our system dynamics models. We begin with the variables associated with the stock-and-flow model of basic operations, which was diagrammed in Figure 2. The stock and rate variables are described in Table A1. Within this table, we use the following logistic function to transform the volume and quality of service variables so that they would follow the law of diminishing marginal utility, which mirrors what was observed with the real data:

$$L\left( x \right)=\frac{1-exp(-2x)}{1+exp(-2x)}$$

Here, $x$ is the variable that is transformed (e.g., volume).

***Table A1:*** *Stock and rate variables used in the system dynamics model of basic operations at the health facility level in Afghanistan*

| **Variables (abbreviation used in Figures 2 and 4)** | **Type** | **Mathematical Definition** | **Notes** |
| --- | --- | --- | --- |
| Revenue (*R*) | stock | derivative = *rR* – *rRQ*,  initial value = 0 | The value of 0 corresponds to the revenue required to maintain the facility at baseline levels of quality and volume |
| Quality of service (*Q*) | stock | derivative = *rRQ* – *rQ*,  initial value = 0 | The value of 0 corresponds to the average quality at baseline |
| Volume of service (*V*) | stock | derivative = *rV*,  initial value = -0.5 | The value of 0 corresponds to the average volume at baseline |
| Recovery rate of revenue (*rR*) | rate | 0.3**L*(*V*) |  |
| Conversion rate of revenue to quality (*rRQ*) | rate | 0.2**R* |  |
| Depletion rate of quality (*rQ*) | rate | 0.3**L*(*V*) |  |
| Recovery rate of volume (*rV*) | rate | 0.1**L*(*Q*_delayed_) | For the variable *Q*_delayed_ we use the value of *Q* with a 3-month delay. |

Next, we describe the variables associated with the stock-and-flow model of the P4P impact on basic operations, which was diagrammed in Figure 4. The auxiliary and rate variables included in this stock-and-flow model are described in Table A2, with parameter values listed in Table A3 for each of the scenarios described in Table 1 in the main body of the paper. The time-dependent variable $P4P$ for the performance bonus allocation at the facility level is determined by the volume of service *V*_delayed_ with a delay given by the parameter *P4P delay* (Table A3, column 3). It has a lower bound of zero because *P4P* is a variable representing the performance bonus and is never negative (so that the bonus can never become a punishment).Gaming *G* at the facility level is modeled as the combined effect of baseline gaming, *G*_0_, and gaming that arises from P4P bonuses (Table A2, row 5). Motivation *M* at the facility level is the combination of the positive effects of P4P bonuses and the negative effects of gaming; hence the corresponding variables are added to and subtracted from the baseline level of motivation *M*_0_ (Table A2, last row).

The differential equations that govern the interrelated flows into and out of the revenue (*R*), quality (*Q*) and volume (*V*) stocks are as follows:

$$\frac{dR}{dt}=0.3\cdot L\left( V \right)-0.2R\cdot{1.3}^{M}+P4P$$

$$\frac{dQ}{dt}=0.2R\cdot{1.3}^{M}-\left\{ \begin{matrix} 0.3\cdot L\left( V \right)\cdot{1.1}^{G}/{1.3}^{M} & \mathrm{if} V>0 \\ 0.3\cdot L\left( V \right)\cdot{1.3}^{M}/{1.1}^{G} & \mathrm{otherwise} \end{matrix} \right.$$

$$\frac{dV}{dt}=\left\{ \begin{matrix} 0.1\cdot L\left( Q_{\text{delayed}} \right)\cdot{1.3}^{M} & \mathrm{if} Q_{\text{delayed}}>0 \\ 0.1\cdot L\left( Q_{\text{delayed}} \right)/{1.3}^{M} & \mathrm{otherwise} \end{matrix} \right.$$

Other relevant information about these variables is shown in Table A2 below.

***Table A2:*** *Auxiliary and rate variables used for modeling impact of P4P bonuses on operations at the health facility level in Afghanistan*

| **Variables** | **Type** | **Definition** | **Notes** |
| --- | --- | --- | --- |
| *P4P factor* | constant | (see Table A3, column 2) | Percentage of health workers in a facility that received P4P |
| *P4P* | rate | *P4P factor* * *L*(*V*_delayed_) | This is set to zero if the result in column 3 is negative |
| Baseline gaming, $G_{0}$ | constant | (see Table A3, column 5) | Baseline gaming, i.e., the initial degree of gaming before the P4P bonuses. |
| Gaming, $G$ | auxiliary | $G_{0}+{1.1}^{P4P}-1$ | $G=0$ corresponds to the absence of gaming |
| Baseline motivation, $M_{0}$, | constant | (see Table A3, column 4) | Baseline motivation, i.e., the initial degree of motivation before the P4P bonuses. |
| Extrinsic Motivation, $M$ | auxiliary | $M_{0}+P4P-G$ | $M=0$ corresponds to the absence of extrinsic motivation |

**Formal model validation process**

The model validation process followed several iterative steps and the formal aspects have been organized based on a scheme proposed by Barlas (1996) below.

**Direct structure test**

***Conceptual confirmation:*** *identifying elements in the real system that correspond to the parameters of the model (Barlas, 1996)*

In our initial iteration of the model, we confirmed each element of the model (and the way those elements were parameterize) with experts with knowledge about health services delivery at the primary health level, and implementation of the P4P intervention in Afghanistan. We did not include gaming in the model initially. However, as we proceeded with validation testing, we found in the behavior sensitivity test that the P4P bonuses increased volume of services more than was observed in the real system. We returned to the conceptual confirmation test and noted that gaming and its ill effects has been reported in other similar contexts (Glasziou et al. 2012; Woolhandler et al. 2012). Hence, we felt that the model failure in the behavior sensitivity test can be traced back to failure in the conceptual confirmation test. In the next iteration of the model, we included gaming and its influences on bonus payments and motivation. This model passed both the conceptual confirmation and behavior sensitivity tests.

**Structure-oriented behavior tests**

***Phase relationship test:*** *checking if phase relationships obtained from the model agree with those that are observed/expected from the real system (Barlas, 1996)*

We expect that high quality would result in increases in volume, but an overshoot in volume would exceed the service capacity of the system, leading to decreased quality. The decrease in quality, in turn, would lead to volume that would eventually go below service capacity. With low volume, the service workers would have the time and energy to increase quality and the cycle would repeat again. In this cycle, there is a phase lag between quality and service, and this is consistently exhibited in all the cases shown in Figures 5 and 6. This phase relationship is most pronounced during the initial months of each simulation before the system reaches equilibrium.

***Extreme-condition test:*** *assigning extreme values to selected parameters and comparing the model-generated behavior to the observed/anticipated behavior under the same extreme condition (Barlas, 1996)*

We ran three extreme-conditions tests: *High motivation*. In this case, we expect high quality of services, which would lead to high demand for these services and hence high volume. At the extreme case where the motivation is exceptionally high, quality would not degrade, even when volume is high. This behavior is shown in Figure 5c. *Extensive gaming*. In this case, the P4P bonuses would not be associated with the true volume of services to clients. Hence, there would be decreased extrinsic motivation, which would negatively impact quality. Volume would also fall. Figure 5d shows a rapid reduction of quality and volume for a simulated system with extensive gaming, which is consistent with what is expected. *High initial revenue*. In the results shown in Figures 5 and 6, it is assumed that the revenue stock accumulates starting from zero at the beginning of the simulation. If, instead, we begin with a large revenue stock, then there would be a large initial revenue flow to the workers, which would make them highly motivated, resulting in high quality and volume. However, as the revenue stock is depleted, the revenue flow and motivation would eventually become reduced, leading to reductions in quality and volume. This behavior was observed when the model was run with a high initial revenue.

***Behavior sensitivity test:*** *determining those parameters to which the model is highly sensitive and asking if the real system would exhibit similar high sensitivity (Barlas, 1996)*

Here we show results of a sensitivity analysis that compares the impact of the P4P intervention under the various scenarios described in the main body of the paper. The basic research question in this analysis is: Using the P4P-only scenario as the baseline, what is the required percentage of the P4P bonuses under the other scenarios that would yield the same level of performance? We use the average of volume and quality of service for the performance outcome in this sensitivity analysis. The last column of Table A3 shows the sensitivity results for the scenarios listed in the first column. For instance, under scenario 3d (bonus delay of 3 months), the P4P bonus would have to be increased to 139% of the original level to counteract the negative effects of the 3-month delay in the bonus. Another way to state this is that if there if there were two facilities, the first with no bonus delay and the second with a bonus delay of 3 months, and if the goal was to have the same level of quality and volume of service in the two facilities, then the second facility would need P4P bonuses that is 139% (39% higher) of the P4P bonuses in the first facility. The negative effect of gaming is worse: under scenario 3b (low-level gaming), the P4P bonuses in the facility with low-level gaming would need to be 310% (210% higher) of the P4P bonuses in the facility without gaming to achieve equal performance.

***Table A3:*** *Sensitivities of the P4P bonus levels for each scenario of basic operations at the facility level.*

| **Scenarios** | **Parameter details** | | | | **Percentage of P4P bonus needed to attain the performance of the P4P-only scenario** |
| --- | --- | --- | --- | --- | --- |
|  | **P4P factor** | **P4P**  **delay** | *M*_0_ | *G*_0_ |  |
| 1. Baseline | 0 | 0 | 0 | 0 |  |
| 2. P4P only | 0.2 | 0 | 0 | 0 | 100% |
| 3a. Motivation | 0.2 | 0 | 0.3 | 0 | 48% |
| 3b. Low-level gaming | 0.2 | 0 | 0 | 0.2 | 310% |
| 3c. High-level gaming | 0.2 | 0 | 0 | 0.8 | * |
| 3d. Bonus delay | 0.2 | 3 | 0 | 0 | 139% |
| 3e. Low bonuses | 0.081 | 0 | 0 | 0 | n/a |
| 4a. Equal allocation | 0.2 | 3 | 0.1 | 0.3 | 411% |
| 4b. Proportionate to salaries | 0.2 | 3 | 0.05 | 0.8 | * |
| 4c. Proportionate to services | 0.2 | 3 | 0.3 | 0.05 | 110% |

**Performance of at the level of the P4P-only scenario is unattainable for any level of P4P bonus.*

Under scenarios 3c and 4b, there does not exist a level of P4P bonus that would produce the same level of performance as the P4P-only scenario. This is primarily due to the insurmountable negative effects of high-level gaming. To illustrate this, we examine the effects of gaming for several levels of the baseline gaming parameter, *G*_0_. The results are plotted in Figure A1, in which the horizontal axis corresponds to the level of gaming and the vertical axis correspond to the last column of Table A3, the required percentage of P4P bonus that would result in the same level of performance as the facility without gaming. The points at *G*_0_ = 0 and *G*_0_ = 0.2 are identical to scenarios 2 (P4P only with no gaming) and 3b (low-level gaming). Hence, the required percentages of P4P bonuses, 100% and 310%, respectively, that are plotted in Figure A1 are the same as the results shown in Table A3. It can be seen in the figure that at gaming levels between 0 and 0.2, the required percentages of P4P bonus has a nearly linear relationship with the level of gaming. At higher levels of gaming, however, the required percentages of P4P bonus begins to increase exponentially. The figure indicates that when baseline gaming approaches *G*_0_ = 0.26, then there is no amount of P4P bonus that can counteract the negative effects of such high levels of gaming. In other words, *G*_0_ = 0.26 is the threshold of insurmountable gaming. This explains the result of scenario 3c, in which the baseline gaming of *G*_0_ = 0.8 exceeds this threshold. Scenario 4b has a similar result because of the high level of gaming associated with this strategy for distributing bonuses, as explained in the main text.


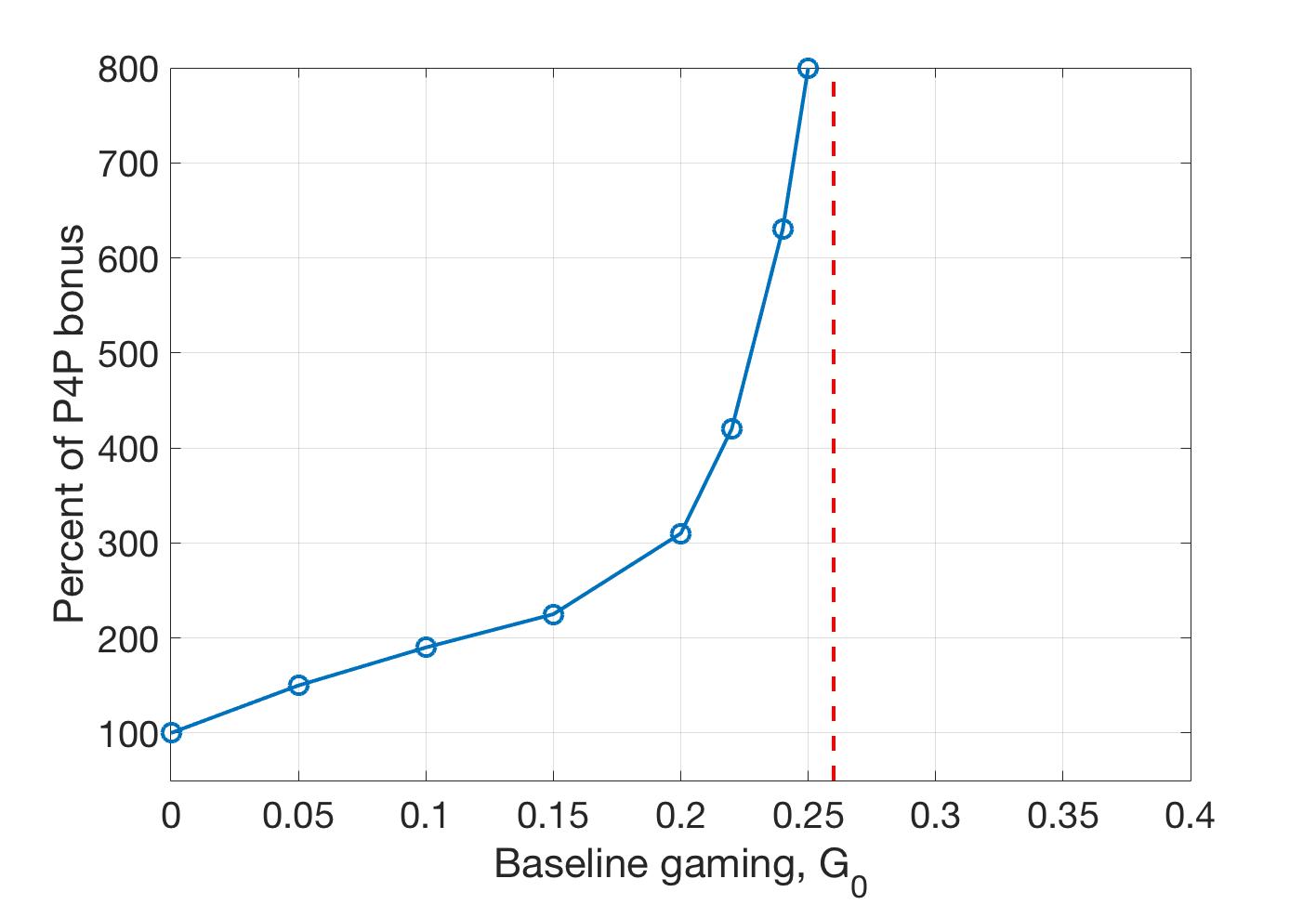


***Figure A1:*** *Required percentages of P4P bonus needed to match the same level of performance as the facility without gaming.*

*Summary of sensitivity tests:* Our behavior sensitivity test results are summarized in Table A3. The last column of this table is an indicator of the sensitivity of performance with respect to the P4P bonus. Here, small percentage values correspond to high sensitivity and large values correspond to low sensitivity. The model passes the behavior sensitivity tests in several dimensions: *Gaming*. If there is even a small amount of gaming (row 3b), then there is a reduced sensitivity with respect to the P4P bonus because the service workers can no longer clearly see the relationship between volume of service and reward. This is reflected by the fact that a larger P4P bonus (310%) is needed to attain a target performance level as compared with the baseline system (row 2) with no gaming. *Motivation.* Increased motivation (row 3a) increases the sensitivity to the P4P bonus because only 47% of the baseline bonus is needed to achieve the same target performance level. *Bonus delay*. The bonuses are eventually received by workers, but they are inconvenienced by the delay. While we expect decreased sensitivity, we do not expect the amount of decrease to be as substantial as for gaming, which has a direct impact on motivation. This is indicated by the result in the table (row 3d) where 139% of the P4P bonus is needed to attain the target performance level.

**Model revisions in response to validation test results**

Two major iterations of the model were needed during validation testing, as explained under the direct structure test commentary above.
